# Neuropsychological aspects of the patient’s personality in the context of psychosomatic experience

**DOI:** 10.3389/fpsyt.2025.1596321

**Published:** 2025-07-02

**Authors:** Robert Krause, Tomáš Forgon

**Affiliations:** Constantine The Philosopher University in Nitra, Slovakia Constantine The Philosopher University in Nitra, Nitra, Slovakia

**Keywords:** personal variables, psychosomatic experience, patient, clinical symptom, neuropsychology

## Abstract

The primary objective of this study was to examine the relationship between personal variables, as measured by the NEO-PI-R, and psychosomatic symptoms, assessed through the PSS, from a neuropsychological perspective. The secondary aim was to evaluate the frequency of various psychosomatic symptoms and assess the extent to which participants experience these symptoms. This online study included participants from across Slovakia (n = 222, M = 34.0, SD = 9.49). Participants completed the Big Five personality questionnaire (NEO-PI-R) and the Scale of Psychosomatic Symptoms (PSS). Analysis revealed significant relationships between conscientiousness and overall health perception (β=−1.19∗∗), with conscientiousness positively correlating with the intensity of pseudoneurological (β=.21∗∗), cardiovascular (β=.15∗), and musculoskeletal symptoms (β=.15∗). Neuroticism was significantly related to overall health perception (β=.19∗∗), problem frequency (β=−.19∗∗), and the extent of health-related suffering (β=−.15∗), as well as the frequency of respiratory (β=−.14∗) and gastrointestinal issues (β=−.16∗), pain-related problems (β=−.18∗∗), and the intensity of gastrointestinal symptoms (β=−.14∗). Extraversion, openness, and agreeableness did not show significant relationships with psychosomatic symptoms (p>0.05). The majority of participants (56%) did not seek medical attention, while 44% did. Seventy percent had no medical diagnosis, while 30% reported a diagnosis from a healthcare professional. The most frequently reported symptoms were fatigue (M=2.69,SD=0.835) and back pain (M=2.32,SD=0.950).

## Introduction

Heart palpitations, chest pressure, muscle tightness, as well as impaired breathing, dizziness, dry mouth, and nausea are bodily symptoms signaling that something is happening within our body. However, are these symptoms of somatic origin, or are they merely consequences of our mental processes? Answering this question often requires cooperation among numerous specialists, who first need to rule out life-threatening variables and make an accurate diagnosis. Wise (1) argues that biological, psychological, and social factors interact significantly, and the expert’s task is to first identify which factor triggers the entire process described by the patient as discomfort. Bakal et al. (2) claim that despite advances in medical science, certain psychosomatic symptoms and diseases remain unknown within the field of medicine, especially concerning various psychobiological mechanisms. Although the cause may sometimes be known and the consequence observed, the treatment process necessitates a multidisciplinary approach from various experts. Simon et al. (3), for example, compiled a list of symptoms that are somatic in nature but often arise as a result of stress reactions in the body. These were categorized into four groups based on which organs are affected: gastrointestinal (abdominal pain, vomiting, unpleasant taste in the mouth, throat tightness, bloating), neurological (headache, dizziness, blurred vision, ringing in the ears, tingling in limbs, goosebumps), musculoskeletal (back pain, joint pain, limb pain, difficulty walking, weakness), and vegetative (chest pain, shortness of breath, heart palpitations, trembling). Simon et al. (3) also observed that when considering the significance of individual symptoms, in cases of accumulated stress, the most frequently occurring symptoms are muscle weakness, headache, heart palpitations, and impaired breathing. Due to unmet needs, the autonomic nervous system, as well as hormones primarily controlled through the hypothalamus-pituitary axis and endocrine glands, behave differently. The interplay between psychological characteristics and physical health has long been a subject of interest in health psychology and behavioral medicine. Among the various psychological constructs investigated, personality traits, representing relatively stable patterns of thoughts, feelings, and behaviors, have emerged as potentially significant predictors and correlates of physical health outcomes, including the manifestation of psychosomatic symptoms. The Five-Factor Model (FFM), or the Big Five, stands as a widely accepted and empirically supported taxonomy of personality traits, encompassing Openness to Experience, Conscientiousness, Extraversion, Agreeableness, and Neuroticism. The Diagnostic and Statistical Manual of Mental Disorders (DSM-V) classifies somatic symptom disorders as those with one or more physical symptoms that cannot be explained by the patient’s medical condition. In other words, these symptoms are often accompanied by excessive thoughts and emotions, which cause significant distress (4). Research (5, 6) has shown that, for example, higher levels of neuroticism negatively affect the quality of psychological experiences, and individuals with higher levels of neuroticism also struggle more with managing various mental difficulties or problems. Additionally, individuals with higher levels of extroversion tend to have a higher quality of life and a lower risk of developing mental disorders. As for other personality factors from the Big Five model, no explicit results have been found regarding their association with quality of life (7). Increased levels of neuroticism are associated with a tendency to experience more negative emotions such as anxiety, sadness, and anger, which also indicates a reactivity of the limbic system, which may result in more frequent activation of the hypothalamic-pituitary-adrenal (HPA) axis, leading to increased production of cortisol (the stress hormone). Chronically elevated cortisol levels can contribute to a variety of psychosomatic symptoms, such as headaches, digestive problems, insomnia, and weakened immunity. Conscientiousness is associated with better prefrontal cortex function, which may lead to more effective stress management and a healthier lifestyle, thus reducing the likelihood of psychosomatic symptoms (8). Research by Wilson et al. (9)Christensen et al. (10), and Weiss and Costa (11) has suggested that, with regard to health and disease associations, personality variables such as agreeableness, conscientiousness, and openness appear to be insignificant. Therefore, in this study, we have decided to explore these personality variables in relation to psychosomatic variables.

### Research problem

The primary objective of this final thesis was to verify the direction and strength of the relationship between personality variables and psychosomatic symptoms. Reflecting on the goal of our work, we formulated the following research question: What is the relationship between personality variables and psychosomatic experiences?

### Research focus

Patient management in the context of psychosomatic experiences involves several specialists, as the cause and effect are not always the same. It is essential to rule out not only physical but also psychological causes for the onset of specific physical symptoms. In this context, our research explored the role of personal variables—neuroticism, extraversion, openness, conscientiousness, and agreeableness—in the management of patients with psychosomatic experiences. Research by Aben et al. (12) highlighted that low extraversion is often associated with lower quality of life. This was further corroborated by Aben et al. (12), who found that low extraversion contributes to the development of depression, a finding supported by Kim et al. (13), who observed a decreased psychological quality of life in individuals with low extraversion. The studies by Aben et al. (12), Kootker et al. (14), and Storror and Byrne (15) further revealed that higher neuroticism predicts the onset of depression, anxiety, and also influences overall psychological and physical well-being, which in turn affects the patient’s perception of health problems. Additionally, research by Amichai-Hamburger and Vinitzky (16) pointed out that openness to new experiences positively correlates with expressiveness, which may, in some situations, explain more emotionally than rationally driven behaviors. Caspi et al. (17) found that individuals with higher levels of agreeableness had a lower likelihood of developing various illnesses.

### Research aim and research questions

The primary aim of this thesis was to verify the direction and strength of the relationship between personality variables (obtained from the NEO-PI-R) and psychosomatic symptoms (obtained from the PSS). The secondary aim was to verify the frequency of experiencing various psychosomatic symptoms and to determine the extent to which participants suffer from specific symptoms. In formulating the objectives of the study, as well as the research questions, we considered the research by Widiger and Mullins-Sweatt (18), who pointed out that the domains of the Big Five provide valuable insights for both healthy and clinical populations. Reflecting on conflicting findings (5–7, 9, 10, 12, 14, 19, 20), we decided not to operate with hypotheses but to formulate research questions. The research questions were designed to provide answers that would help achieve the defined goal of the study. We formulated the following research questions:

RQ1: What is the relationship between personality variables (neuroticism, extraversion, openness, agreeableness, conscientiousness) and psychosomatic symptoms?
RQ2: What percentage of individuals use painkillers?
RQ3: What percentage of individuals had to visit a doctor due to health problems?
RQ4: What percentage of individuals have a diagnosis of asthma, diabetes, or a diagnosed allergy?
RQ5: Which psychosomatic symptoms appear most frequently? RQ6: Which psychosomatic symptoms appear least frequently?
RQ7: Which psychosomatic symptoms cause the most distress for individuals? RQ8: Which psychosomatic symptoms cause the least distress for individuals?

## Research methodology

### General background

In the presented thesis, we focused on the relationship between personality variables and the psychosomatic experiences of patients. In the first step, we selected research methodologies that are both valid and reliable, enabling us to answer the research question. We decided to use the Psychosomatic Symptom Scale by Anita Vulić – Prtorić (21) and a specially adapted Big Five questionnaire, which was used in the research by Terracciano et al. (22). Subsequently, we transformed the questionnaires into an online format and, utilizing the services of Survio (www.survio.com), which offers advanced data collection features, we distributed the questionnaires to various target groups. After the questionnaires were completed, the data were subjected to statistical analysis, and the results are presented in the section dedicated to research findings.

### Sample

The research sample (n = 222) consisted of participants from across Slovakia, ranging in age from 15 to 71 years (M = 34.0, SD = 9.49). The sample included participants with varying levels of education, including primary education (n = 2), secondary education (n = 67), and higher education (n = 143). Participants were selected by purposive sampling and were made up of people from an internal database who had previously expressed interest in participating in psychological research.

### Instrument and procedures

Research data were obtained using the following questionnaires:

## NEO personality inventory, revised

In this study, we utilized a modified version of the Big Five questionnaire, as employed by Terracciano et al. (22). The internal consistency of this measurement instrument, as indicated by Cronbach’s alpha for the five-factor scales—Neuroticism (α = 0.74), Extraversion (α = 0.73), Openness (α = 0.50), Agreeableness (α = 0.64), and Conscientiousness (α = 0.65)—was found to be acceptable. The questionnaire consists of 30 items, with participants required to indicate their level of agreement or disagreement with each statement on a five-point Likert scale. This instrument assesses five personality factors: Neuroticism, Extraversion, Openness, Agreeableness, and Conscientiousness.

## Psychosomatic symptoms scale

The Psychosomatic Symptoms Scale (PSS) was developed by Anita Vulić – Prtorić (21) and consists of 35 somatic symptoms categorized into the following domains: Pseudoneurological (vertigo, loss of balance, lump in the throat, double vision, blurred vision, sudden loss of vision, sudden hearing loss, fainting, sudden memory loss); Cardiovascular (rapid heartbeat, chest pain, excessive sweating); Muscular (muscle tension, muscle weakness); Respiratory (breathing difficulties, feeling of suffocation, cold-related symptoms such as sore throat and cough); Gastrointestinal (nausea, abdominal cramps—excluding menstrual pain, diarrhea, vomiting, bloating, loss of appetite, intolerance to certain foods, constipation, heartburn); Dermatological (skin rash, itching/red skin, acne or pimples); Pain and Weakness (headache, back pain, lack of energy/fatigue, high body temperature, joint pain, pain in hands/legs). The scale also includes items that assess additional relevant information, such as the subjective experience of painful symptoms, with the severity of their presence aligned with DSM-V criteria. Participants are required to indicate the frequency of each symptom on a 4-point scale and the extent to which a given symptom troubles them on a 3-point scale.

### Data analysis

Statistical analysis was conducted using SPSS Statistics 20. Data were analyzed not only at the level of descriptive statistics but also through correlation analyses. In the initial step, we performed reverse scoring for items in the NEO-PI-R that were formulated in the opposite direction. Subsequently, we calculated the average scores for the NEO-PI-R factors and separately computed the mean scores for the PSS factors— one for the frequency of experienced symptoms and another for the intensity of distress caused by these symptoms. Additionally, we assessed reliability using McDonald’s omega coefficients for each scale/symptom. As part of the evaluation, we also examined the percentage distribution of experienced psychosomatic symptoms.

## Research results

We found significant relationships between conscientiousness and overall health perception (b = -1.19**, indicating that the more conscientious individuals are, the poorer their health is perceived) (**Table 1**). Additionally, conscientiousness positively correlated with the intensity of distress associated with pseudoneurological symptoms (b = .21**), cardiovascular symptoms (b = .15*), and muscular symptoms (b = .15*), suggesting that more conscientious individuals experience greater distress regarding issues in these domains.

**Table 1:** Relationship between personality variables and Individual psychosomatic symptoms.

Variable	1.	2.	3.	4.	5.	6.	7.	8.	9.	10.	11.	12.	13.	14.	15.	16.	17.	18.	19.	20.	21.	22.
1. Neuroticism	(.74)																					
2. Extraversion	.15*	(.73)																				
3. Openness	-.02	-.11	(.50)																			
4. Agreeableness	.16*	.17*	.09	(.64)																		
5. Conscientiousness	.15*	.03	.13	.14*	(.65)																	
6. Perceived Overall Health	.19**	.10	-.05	.09	- .19**	—																
7. PSS Symptom Frequency	- .19**	-.05	.05	.03	.08	- .43***	(.89)															
8. PSS Symptom Intensity	-.15*	-.03	.04	.01	.13	- .42***	.89***	(.89)														
9. Pseudoneurological Symptom Frequency	-.06	-.06	.05	.04	.11	- .30***	.69***	.63***	(.67)													
10. Pseudoneurological Symptom Intensity	.02	-.03	.00	.02	.21**	- .34***	.58***	.66***	.85***	(.69)												
11. Cardiovascular Symptom Frequency	-.10	-.03	.06	.08	.08	- .26***	.69***	.63***	.56***	.50***	(.50)											
12. Cardiovascular Symptom Intensity	-.11	-.02	.13	.08	.15*	- .27***	.64***	.72***	.48***	.53***	.85***	(.59)										
13. Muscular Symptom Frequency	-.12	-.08	-.03	.00	.08	- .27***	.66***	.58***	.45***	.34***	.43***	.41***	(.69)									
14. Muscular Symptom Intensity	-.13	-.01	.02	.02	.15*	- .30***	.62***	.62***	.43***	.41***	.40***	.45***	.87***	(.64)								
15. Respiratory Symptom Frequency	-.14*	-.06	.09	.03	-.02	-.21**	.56***	.53***	.33***	.28***	.41***	.41***	.25***	.30***	(.65)							
16. Respiratory Symptom Intensity	-.13	-.04	.07	.01	-.02	-.17*	.55***	.63***	.35***	.35***	.38***	.45***	.28***	.33***	.87***	(.65)						
17. Gastrointestinal Symptom Frequency	-.16*	-.01	.06	-.04	.01	- .33***	.84***	.80***	.53***	.46***	.49***	.48***	.54***	.52***	.45***	.48***	(.75)					
18. Gastrointestinal Symptom Intensity	-.14*	.03	.03	-.06	.06	- .32***	.75***	.82***	.48***	.47***	.44***	.49***	.46***	.47***	.36***	.45***	.90***	(.78)				
19. Dermatological Symptom Frequency	-.13	-.02	-.07	-.00	.08	-.20**	.58***	.52***	.24***	.18**	.33***	.29***	.30***	.27***	.25***	.26***	.35***	.33***	(.73)			
20. Dermatological Symptom Intensity	-.09	-.05	-.07	-.01	.09	-.16*	.51***	.58***	.18**	.16*	.29***	.32***	.27***	.26***	.27***	.34***	.34***	.39***	.87***	(.75)		
21. Pain Frequency	- .18**	-.09	.02	.09	.06	- .43***	.79***	.63***	.45***	.36***	.44***	.38***	.50***	.45***	.42***	.38***	.57***	.45***	.38***	.31***	(.59)	
22. Pain Intensity	-.12	-.07	-.05	.01	.03	- .40***	.68***	.77***	.39***	.44***	.38***	.45***	.49***	.50***	.36***	.46***	.53***	.52***	.35***	.40***	.75***	(.55)

The omega coefficients for McDonald’s are reported in parentheses. * p <.05; ** p <.01; *** p <.001.

Our results also revealed statistically significant relationships between neuroticism and overall health perception (b = .19**), as well as with the total frequency of reported problems (b = -.19**) and the overall intensity of health-related distress (b = -.15*). Moreover, we observed statistically significant associations between neuroticism and the frequency of respiratory problems (b = -.14*), gastrointestinal problems (b =-.16*), pain-related issues (b = -.18**), and the intensity of gastrointestinal symptoms (b = -.14*). In simple terms, these findings suggest that higher levels of neuroticism among participants were associated with better reported health and lower levels of pain.

Among the other personality factors (extraversion, openness, and agreeableness), no statistically significant relationships with psychosomatic symptoms were observed (p > 0.05). Furthermore, statistically significant associations were found among the various psychosomatic symptoms themselves, indicating that if an individual experiences difficulties in one domain, they are likely to experience challenges in other areas as well.

We found that the majority of participants (52.3%) rated their health as very good, with none rating their health as poor. Additionally, 140 participants (63%) reported not feeling the need to take medication, while 81 participants (37%) indicated a need for medication. Furthermore, 123 participants (56%) did not have to visit a doctor due to their health issues, whereas 98 participants (44%) did seek medical attention for their symptoms. The results further indicate that 155 participants (70%) were not diagnosed with any disease, while 66 participants (30%) had received a diagnosis from a specialist.

Moreover, our findings revealed that the most frequently reported symptom was fatigue (M = 2.69, SD = 0.835). The second most frequent symptoms were back pain (M = 2.32, SD = 0.950) and a bloated stomach (M = 2.10, SD = 0.884), followed by acne (M = 1.95, SD = 0.950) and headache (M = 1.92, SD = 0.609). In contrast, the least common symptoms in our study were hearing loss (M = 1.00, SD = 0.067), loss of vision (M = 1.03, SD = 0.221), fainting (M = 1.05, SD = 0.208), double vision (M = 1.11, SD = 0.326), and memory loss (M = 1.12, SD = 0.375).

Additionally, our analysis showed that participants experienced the greatest level of distress from fatigue (M = 2.30, SD = 0.663), followed by back pain (M = 2.00, SD = 0.684), a bloated stomach (M = 1.94, SD = 0.772), and acne (M = 1.76, SD = 0.77), as well as headache (M = 1.70, SD = 0.656). Conversely, the least distress was associated with hearing loss (M = 1.01, SD = 0.150), followed by loss of vision (M = 1.04, SD = 0.250), double vision (M = 1.10, SD = 0.361), a sensation of choking (M = 1.15, SD = 0.488), or vomiting (M = 1.15, SD = 0.441).

## Discussion

Our results revealed statistically significant relationships between conscientiousness and overall health perception, indicating that individuals who are more conscientious tend to perceive their health as poorer compared to those who are less conscientious. We also found that conscientiousness positively correlated with the intensity of distress associated with pseudoneurological, cardiovascular, and muscular symptoms, suggesting that more conscientious individuals experience greater distress regarding issues in these domains. This finding largely supports the results of Raynor and Levine (23), who observed that highly conscientious individuals not only engage more in preventive health behaviors but also tend to be more concerned about their health. It appears that conscientiousness functions as a preventive factor, contributing to a healthier lifestyle; however, it may also lead to a higher degree of psychosomatization, as these individuals are more attentive to and troubled by their health status. Although Murphy et al. (24) contend that conscientiousness reduces the tendency to expose oneself to stressful experiences, our research indicates that the level of psychosomatization is higher among more conscientious individuals than among those who are less conscientious. We explain this phenomenon by suggesting that while more conscientious individuals are proactive in adopting preventive measures - which contributes to better overall health - they may concurrently experience increased psychosomatic distress.

In addressing our research question (RQ1), we also found statistically significant associations between neuroticism and overall health perception, the total frequency score of health problems, as well as the overall intensity of health-related distress among participants. Moreover, a statistically significant relationship was observed between neuroticism and the frequency of respiratory and gastrointestinal issues, as well as with pain-related problems and the intensity of gastrointestinal symptoms. These findings contradict previous research by Aben et al. (12)Kootker et al. (14), and Storor and Byrne (15), which reported that higher levels of neuroticism predict the onset of depression and anxiety and adversely affect overall psychological and physical well-being, thereby influencing the patient’s perception of health problems. In contrast, our study found that higher levels of neuroticism were associated with better self- reported health and lower levels of psychosomatic pain. It appears that the manifestations of neuroticism have a greater impact on objective health issues than on the degree of psychosomatization. Furthermore, it was observed that inappropriate reactions, restlessness, and hypersensitivity - characteristic of elevated neuroticism - do not influence the extent to which an individual is troubled by symptoms that they do not perceive as present. Regarding the other personality factors (extraversion, openness, and agreeableness), no statistically significant relationships with psychosomatic symptoms were found. Although previous research by Aben et al. (12), Ross et al. (25), and Kim et al. (13) indicated that lower levels of extraversion are associated with diminished quality of life, this association was not evident in our study in the context of psychosomatization and distress over specific symptoms. Similarly, our research did not confirm Booth-Kewley and Vickers’ (26) findings, which suggested that agreeableness is positively correlated with health- promoting behavior. Instead, our results align more closely with those of Mirnics et al. (6), who also did not find a statistically significant relationship between openness and the perception of health and illness. It appears that, in terms of psychosomatic experiences, neuroticism and conscientiousness play the most significant roles, whereas extraversion, openness, and agreeableness are less influential. This conclusion is further supported by the significant correlations we observed among various psychosomatic symptoms. We agree with Rosmalen et al. (8), that neuroticism is associated with a tendency to experience more negative emotions such as anxiety, sadness, and anger, which also indicates a reactivity of the limbic system, which may result in more frequent activation of the hypothalamic-pituitary-adrenal (HPA) axis, leading to increased production of cortisol (the stress hormone). In practice, this means that if an individual experiences difficulties in one domain, they are likely to encounter challenges in other areas as well. Such findings suggest that psychosomatization is not confined to a single symptom but rather manifests as a constellation of symptoms that tend to accumulate. In real-world terms, an individual who feels unwell may experience heart palpitations, excessive sweating, blurred vision, headaches, or muscular weakness. These interrelationships among symptoms corroborate the observations of Simon et al. (3), who noted that somatic symptoms—often arising as a consequence of stress responses—tend to cluster. This tendency underscores the importance of conducting a thorough differential diagnosis in patient management, as incongruent symptom associations from a medical perspective may indicate the presence of psychosomatization. Our findings further revealed that the most frequently reported symptom was fatigue, followed by back pain, bloated stomach, acne, and headache. Conversely, the least common symptoms were hearing loss, loss of vision, fainting, double vision, and memory loss.

Our findings indicate that within the framework of psychosomatization, symptoms from various categories tend to occur in combination. This observation corroborates the findings of Wisnivesky et al. (27) and Waszczuk et al. (28), as the evaluation of life outcomes is heavily influenced by an individual’s self- assessment. It is sometimes observed that the difficulties reported by patients may be distorted not only by their conscious, deliberate behavior but also by subconscious mechanisms beyond their control. We found that participants were most troubled by fatigue, back pain, a bloated stomach, acne, and headaches, whereas they were least troubled by hearing loss, loss of vision, double vision, sensations of choking, or vomiting. These results are understandable, as individuals tend to be more concerned about symptoms they have recently experienced. In essence, fatigue, back pain, a bloated stomach, and headaches appear to be psychosomatic symptoms that frequently manifest, often in combination. This suggests that special attention should be paid to such symptom clusters during differential diagnosis, since, as Wise (1) explains, biological, psychological, and social factors interact significantly, and the expert’s initial task is to determine which factor triggers the patient’s reported difficulties. Once the underlying cause of the symptoms is identified by a healthcare professional, the subsequent treatment process can be considerably more effective.

Furthermore, we found that 123 participants (56%) did not need to visit a doctor due to their health issues, whereas 98 participants (44%) did seek medical attention because of the symptoms themselves. We interpret this finding to mean that, although 70.3% of participants rate their overall health as excellent or very good, they still prefer to consult a doctor when specific symptoms arise, to ensure that their condition is truly satisfactory. This behavior aligns with the personality variable of conscientiousness, which appears to play a significant role in health perception. Additionally, our findings are consistent with Bessho and Ohkusa (29), who suggest that if a patient perceives their illness as mild, an alternative to visiting a doctor may be to use over-the-counter medication. Overall, our results indicate that 155 participants (70%) were not diagnosed with any disease, whereas 66 participants (30%) had received a formal diagnosis.

Several limitations of this study must be acknowledged. Firstly, the cross-sectional design prevents us from inferring any causal relationships between personality traits and psychosomatic symptoms. The observed associations may be bidirectional, or influenced by unmeasured third variables. Future research should employ longitudinal designs to explore the temporal precedence of these variables.

Secondly, the reliance on self-report measures introduces the potential for self-report bias, including social desirability. Participants may have responded in a way they perceived as more socially acceptable, potentially skewing the reported levels of both personality traits and symptoms.

Thirdly, the cultural specificity of the Slovak sample limits the generalizability of our findings to other cultural contexts. Personality expression and the manifestation of psychosomatic symptoms can be influenced by cultural norms and values. Future research should investigate these relationships in more diverse populations.

Finally, the use of an adapted version of the NEO-PI-R, rather than the full original version, may have impacted the comprehensiveness and reliability of the personality trait assessment. While adaptations can be necessary for linguistic and cultural relevance, they may also lead to a loss of nuance or changes in the factor structure.

In the context of cultural specificity, it is crucial to consider the findings of Favaretto et al. (30), who emphasized that affective temperaments, which are closely related to personality dimensions such as neuroticism, can manifest differently across various populations and environments. Their synthesis of 30 years of clinical experience and scientific knowledge indicates that cultural factors, social norms, and specific stressors within a given environment can influence how neuroticism is expressed and how it affects health perception and psychosomatic symptoms. For instance, within the Slovak context, there may be specific cultural factors influencing the relationship between neuroticism and health perception, and these factors may differ from those in other countries. Therefore, it is important to interpret our findings with consideration for the cultural context and to examine how culturally specific factors may impact the relationship between personality traits and psychosomatic symptoms.

In summary, monitoring the relationship between personality variables and psychosomatic experiences is meaningful, particularly as increased efforts to promote the prevention of psychosomatization could help reduce the burden on healthcare specialists by addressing symptoms that are predominantly psychosomatic rather than purely physical.

## Conclusions and Implications

In our study, we demonstrated that paying attention to psychosomatic experiences is worthwhile, as we found that conscientiousness and neuroticism play a significant role in the frequency of psychosomatic symptoms. We observed statistically significant correlations among various psychosomatic symptoms, which indicates that individuals prone to psychosomatization tend to experience a cumulative effect - where one symptom leads to another - resulting in a vicious cycle of bodily symptoms that are often merely a consequence of a mental apparatus subjected to excessive stress or tension.

Our findings reveal that the most frequently reported psychosomatic symptoms are fatigue, back pain, a bloated stomach, and headaches. These physical symptoms often lie at the intersection of psychological and somatic factors, which largely accounts for the manifestation of psychosomatization. Consequently, adopting a multidisciplinary approach to differential diagnosis in patient management is both logical and essential, since physical symptoms frequently have their origins in psychological experiences, and the extent of psychological distress is reciprocally influenced by the frequency of somatic symptoms. In some cases, patients become trapped in a cycle of their own physical ailments, expecting that a specialist will not only help them understand and conceptualize their condition but also select an appropriate treatment plan.

Establishing adequate treatment often requires a consultative assessment of the patient from the perspective of various specialists, who should consider the potential presence of psychosomatic symptoms during differential diagnosis. Although the correlations between personality variables and the degree of psychosomatization were not highly significant, it is evident that paying particular attention to conscientiousness and neuroticism is indeed worthwhile.

For further research, it is important to also control for potential intervening variables that could have influenced psychosomatic symptoms (such as lifestyle, current health status, etc.).

## Data Availability

The original contributions presented in the study are included in the article/supplementary material, further inquiries can be directed to the corresponding author/s.
